# Aspirin Use Is Not Associated with the Risk of Metachronous Gastric Cancer in Patients without *Helicobacter pylori* Infection

**DOI:** 10.3390/jcm11010193

**Published:** 2021-12-30

**Authors:** Ji Eun Kim, Tae Jun Kim, Hyuk Lee, Yeong Chan Lee, Hwe Hoon Chung, Yang Won Min, Byung-Hoon Min, Jun Haeng Lee, Jae J. Kim

**Affiliations:** 1 Department of Medicine, Samsung Medical Center, Sungkyunkwan University School of Medicine, Seoul 06351, Korea; happyjinny0706@gmail.com (J.E.K.); tj23.kim@samsung.com (T.J.K.); hwehoon.chung@samsung.com (H.H.C.); yangwon.min@samsung.com (Y.W.M.); jason.min@samsung.com (B.-H.M.); jh2145.lee@samsung.com (J.H.L.); jaej.kim@samsung.com (J.J.K.); 2 Research Institute for Future Medicine, Samsung Medical Center, Sungkyunkwan University School of Medicine, Seoul 06351, Korea; conan_8th@naver.com

**Keywords:** EGC, ESD, metachronous, aspirin, prevention

## Abstract

Introduction: *Helicobacter pylori* (*H. pylori*) eradication can prevent metachronous gastric cancer (MGC) after the performance of an endoscopic resection for early gastric cancer (EGC). However, 50% of infections persist after eradication, and the identification of MGC protective factors is important. The anti-tumor activity of aspirin has been demonstrated, but its efficacy in preventing MGC remains controversial. We evaluated the effect of aspirin on metachronous recurrence in *H. pylori*-negative patients. Methods: A total of 4351 patients were evaluated between January 2007 and December 2016, and 2151 patients who met the inclusion criteria were analyzed. The primary outcome was the cumulative incidence of MGC after an endoscopic resection for EGC. Results: During a 5-year median follow-up (interquartile range, 3.5–6.2), MGC developed in 176 (7.7%) patients, with a cumulative incidence of 89.4% in aspirin users and 92.7% in non-users; this difference was not statistically significant (*p* = 0.64). The duration of aspirin uses and the occurrence of MGC in both groups were not significantly different. There was no significant difference between groups when the duration of aspirin use was categorized into ≤1 year (hazard ratio (HR), 0.64; 0.20–2.01, *p* = 0.45), 1–4 years (HR, 1.35; 0.66–2.76, *p* = 0.41), and >4 years (HR, 1.17; 0.67–2.03, *p* = 0.58). Conclusions: Aspirin use was not associated with a lower risk of MGC in *H. pylori*-negative patients. Further prospective studies are needed.

## 1. Introduction

Endoscopic submucosal dissection (ESD) is a standard treatment for early gastric cancer (EGC); however, this procedure is associated with a risk of recurrence in the native stomach, and regular follow-up endoscopies are required. Metachronous gastric cancer (MGC) is generally defined as gastric cancer that is distantly located from the original EGC that develops at least 1 year after the index ESD [1]. The incidence of MCG after the surgical resection of gastric cancer is lower than that after endoscopic removal [2]. Previous studies have reported a cumulative incidence of postoperative MGC of 0.9–3.0%, and 5-year, 7-year, and 10-year cumulative incidence rates post-endoscopic removal of 9.5%, 13.1%, and 22.7%, respectively, over a median follow-up of 82.2 months [3,4].

The efficacy of *Helicobacter pylori* (*H. pylori*) eradication in preventing gastric cancer recurrence has been demonstrated in randomized controlled trials (RCTs) [4,5], where it has been shown to reduce the incidence of MGC by 50%; however, managing the remaining 50% of cases represents an important clinical challenge. Therefore, there is a clinical need to identify the preventive factors for MGC. 

The first report on the association between aspirin and cancer was in 1971 [6,7,8], and more than 40 studies on aspirin and gastric cancer have been conducted since 2016 [8,9,10,11]. Although the exact pathophysiological mechanism underlying this association has not been identified, it is generally established that aspirin lowers the risk of human tumorigenesis in various types of inflammation-related carcinomas, such as colorectal, gastric, and liver cancer [12,13]. The exact molecular mechanism by which aspirin exerts its anticancer effect has not been clearly elucidated, but it appears to involve a complex mechanism with multiple metabolic pathways [14].

There are also few studies on the effect of aspirin on metachronous recurrence. In the case of *H. pylori* infection, which has a proven protective effect on metachronous recurrence, it is necessary to change the molecule and find the point of no return for infected chronic atrophic gastritis and intestinal metaplasia, compared to a single treatment. It has been suggested that aspirin and nonsteroidal anti-inflammatory drugs have additional effects [15].

Previous studies on the effect of aspirin on metachronous recurrence have provided inconsistent conclusions, which may be due to differences in the dose and duration of aspirin treatment as well as study design, including statistical errors due to retrospective analyses and classification errors from large-scale studies. Therefore, the role of aspirin in metachronous recurrence remains controversial. Since *H. pylori* eradication may protect against metachronous recurrence, there is a possibility of bias due to the presence or absence of antibacterial treatments. Therefore, we aimed to investigate the effect of aspirin alone on metachronous recurrence after ESD in *H. pylori*-negative patients.

## 2. Materials and Methods

### 2.1. Study Design, Settings and Participants

**Figure 1:**
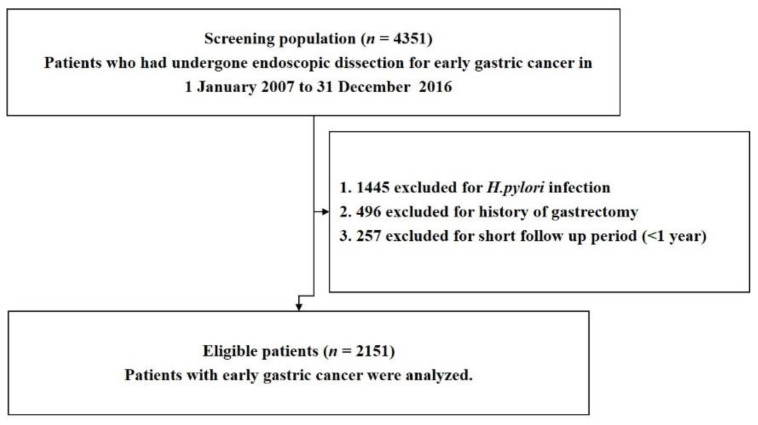
Flowchart of patient enrollment.

This was a retrospective cohort study of patients registered at the Samsung Medical Center, Seoul, South Korea. Patients who underwent endoscopic resection for EGC between January 2007 and December 2016 (*n* = 4351), and who were followed up until 30 April 2020, were included. Patients with (1) *H. pylori* infection, (2) a history of gastrectomy during the study period, and (3) a follow-up duration of less than 1 year were excluded. The number of patients who met the criteria was 2151 (Figure 1). The study protocol was approved by the Ethics Committee of Samsung Medical Center (IRB No. 2021-02-147-001). This study was conducted in accordance with the principles of the Declaration of Helsinki. As this study used only de-identified data routinely collected during hospital visits, the requirement to obtain informed patient consent was waived.

### 2.2. Study Endpoints, Variables, Definitions 

The primary outcome was the incidence of MGC, as confirmed by endoscopic biopsy. At our institution, routine surveillance endoscopy after ESD is performed annually. MGC was defined as GC newly diagnosed at least 1 year after the index date (the date of ESD for EGC). 

Electronic medical records were reviewed to identify patients who were prescribed aspirin during the follow-up period. Aspirin users were defined as those who were prescribed aspirin for ≥30 days. To confirm the relationship between treatment duration and response, aspirin use was categorized into seven groups: no use, >6 months, >1 year, >2 years, >3 years, >4 years, and 5 years of use. To assess trends in the duration of aspirin use, patients were subdivided into four groups: no use, <1 year, 1–4 years, and >4 years of use.

The clinical variables assessed included age, sex, and comorbidities, such as hypertension, diabetes mellitus (DM), myocardial infarction (MI), heart failure (HF), chronic kidney disease (CKD), liver cirrhosis (LC), and cerebrovascular disease (CVD). Tumor size, location, macroscopic type, depth of invasion, lymphovascular invasion, and histologic definition (based on the World Health Organization classification) were investigated to confirm the recurrence of the original EGC’s characteristics.

### 2.3. Statistical Analyses

We computed the sample size required for a time-to-event analysis using the following variables: type 1 error 0.05, power 0.95, hazard ratio 1.0, testing margin 0.5, and event rate, 1.0. We set a hazard ratio of 1 because the association between the use of aspirin and the risk of metachronous gastric cancer is controversial; however, we set the power at a robust 0.95. We subsequently obtained an expected sample size of 2080 [16,17]. Continuous variables were expressed as mean ± standard deviation or median with ranges. Student’s t-test or the Mann–Whitney test were used to compare continuous variables. Categorical variables were expressed as counts with percentages and compared using Fisher’s exact test. The cumulative incidence of MGC was analyzed using Kaplan–Meier curves, and the difference between the curves was tested using the log-rank test. Adjusted hazard ratios (HRs) and 95% confidence intervals (CIs) were estimated using the Cox proportional hazard model to identify the significant factors affecting recurrence. For multivariable analysis, variables with *p* < 0.1 in univariable analysis were selected. In the subgroup analysis, Bonferroni correction was additionally performed. All analyses were considered to be statistically significant at *p*-values of <0.05, and were performed using SAS version 9.4 (SAS Institute, Cary, NC, USA). 

## 3. Results

### 3.1. Baseline Characteristics of the Overall Cohort 

Baseline characteristics are summarized in Table 1. A total of 236 patients were aspirin users and 1915 were non-users. The mean age was 64 years, and 78.9% of the patients were men. The aspirin user group comprised older individuals and exhibited a higher proportion of men. The aspirin user group also had a higher proportion of patients with hypertension, DM, MI, HF, CKD, LC, and CVD. However, there was no difference in tumor characteristics, including size, location, depth of invasion, and histologic differences, except for macroscopic type, between the two groups (Table 1).

### 3.2. Factors Associated with MGC 

**Figure 2:**
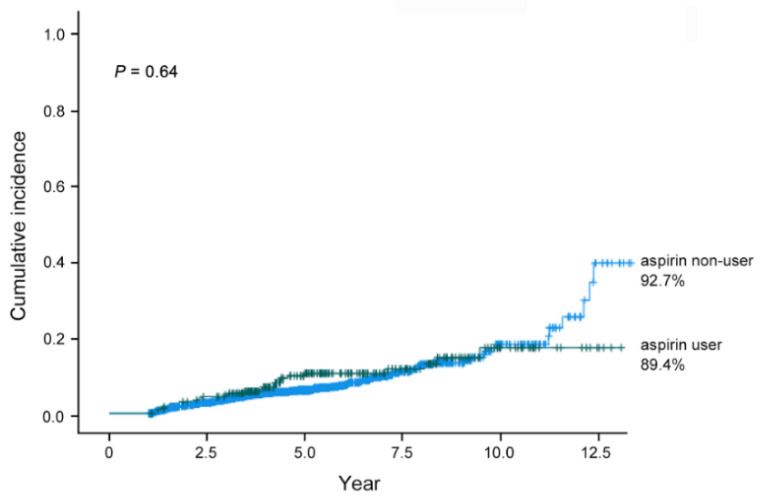
Cumulative incidence of metachronous gastric cancer according to aspirin use.

With a median follow-up of 5 years (interquartile range, 3.5–6.2), 176 (7.7%) patients developed MGC. Except for age, no other clinical variables, including aspirin use, were significantly associated with the cumulative incidence of MGC. The tumor characteristics, including tumor size, location, and macroscopic type, were significant (*p* < 0.1) in the univariate analysis. However, no independent predictors were identified in the multivariate analysis. Marginal differences were observed according to tumor location (*p* = 0.082, Table 2). A subgroup analysis of the factors associated with MGC is shown on Supplementary Materials Table S1, and no significant factor was found. The cumulative incidence of MGC was 89.4% in aspirin users and 92.7% in non-users, but the difference was not statistically significant (*p* = 0.64, Figure 2).

### 3.3. The Effect of the Duration of Aspirin Use on the Occurrence of MGC

We evaluated the association between the duration of aspirin use and MGC in both groups. There was no significant difference between the >6 months (HR, 1.15; 95% CI, 0.73–1.79, *p* = 0.55), >1 year (HR, 1.22; 0.78–1.79, *p* = 0.38), >2 years (HR, 1.26; 0.78–2.01, *p* = 0.34), >3 years (HR, 1.16; 0.69–1.95, *p* = 0.57), >4 years (HR, 1.15; 0.66–2.00, *p* = 0.61), and >5 years of aspirin use groups (HR, 1.01; 0.54–1.86, *p* = 0.98). Similarly, there was no significant difference between the 1 year (HR, 0.64; 0.20–2.01, *p* = 0.45) and 1–4 years of use groups (HR, 1.35; 0.66–2.76, *p* = 0.41) (Table 3).

## 4. Discussion

The association between aspirin use and the development of MGC after ESD remains controversial. In our cohort of *H. pylori*-negative patients who underwent ESD for EGC, aspirin use was not associated with a decreased risk of GC. 

The effect of aspirin use on gastric cancer has been evaluated in several studies. For example, Ihara et al. found that the incidence of intestinal-type gastric cancer was higher in aspirin users than in non-users (0.14%; *p* = 0.004) [18]. The molecular basis of the effects of *H. pylori* eradication and aspirin use on the risk of MGC have also been explored [15]. In another meta-analysis, aspirin use was associated with a reduced incidence of several cancers (odds ratio 0.62; 95% CI, 0.55–0.70; *p* *<* 0.0001). However, most of the included studies were case–control studies that evaluated the incidence of gastric and/or esophageal cancers in Western populations and did not include Asian participants [6]. Aspirin is associated with important adverse events, especially gastrointestinal (GI) bleeding. This was demonstrated in the meta-analysis by Haykal et al., who found that aspirin was not associated with all-cause mortality or cancer incidence, but was significantly associated with major GI bleeding compared to the placebo group (relative risk, 1.85; 95% CI, 1.38–2.48; *p* *<* 0.01) [19].

Aspirin has also been demonstrated to have a favorable effect in patients after radical therapy for various cancers, including esophageal, colorectal, breast, and prostate cancers [20]. The phase three AspECT trial for Barrett’s metaplasia concluded that treatment with high-dose proton-pump inhibitors and 300 mg aspirin, but not aspirin alone, prolongs the time to high-grade dysplasia and esophageal adenocarcinoma [21].

The results of our study are similar to those of two recent large independent population-based cohort studies, which found that low-dose aspirin use was not associated with cancer-specific or all-cause mortality in esophageal and gastric cancers (pooled adjusted HR, 0.94; 95% CI, 0.86–1.02; adjusted HR, 0.95; 95% CI, 0.88–1.02, respectively) [16]. Although the aspirin dose and study methodology in this large-scale study were similar to ours, the latter included English and Scottish cohorts, while ours included Asian patients with gastric cancer and also considered the bleeding risk. An important advantage of our study is that we only included *H. pylori*-negative patients. Patients taking aspirin are likely to have underlying medical conditions. Aspirin is associated with a risk of bleeding due to its antithrombotic action and this risk must be balanced against any potential anticancer effects. 

Among the 1445 patients with *H. pylori* in our study, the cumulative incidence of MGC was 93.2% in aspirin users and 94.2% in non-users, which was not a statistically significant difference (*p* = 0.46, Supplementary Materials Figure S1). At our medical center, patients who are *H. pylori*-positive on follow-up esophagogastroduodenoscopy after ESD receive eradication treatment. Patients may or may not undergo eradication depending on various comorbidities and circumstances, and there are patient groups that undergo second and third round eradication treatments. Furthermore, some may develop *H. pylori*-caused or *H. pylori*-negative cancer. Therefore, further prospective studies on the development of aspirin-related MGC in *H. pylori-positive* groups are needed.

Several studies have investigated the mechanisms by which aspirin affects carcinogenesis [14,22,23]. Aspirin may exert its effect through the Wnt/B-catenin signaling pathway, cyclooxygenase-mediated synthesis, or the AMP-activated protein kinase (AMPK) pathway, where the direct binding of salicylic acid to AMPK results in its activation. These studies suggest that aspirin exerts its anti-tumor effects by activating inflammatory signaling pathways. Furthermore, aspirin can affect various cell types, such as platelets, and epithelial, cancer, stromal, immune, and endothelial cells. Aspirin affects not only the normal parenchymal tissue and tumor cells, but also the stroma and tumor microenvironment [24,25]. However, further studies are required to elucidate the exact pathomechanism through which aspirin exerts its anti-tumor effects. 

This study had some limitations. First, this was a retrospective study of electronic medical records; therefore, selection bias may be present. Furthermore, negative *H. pylori* status was confirmed based on clinical records rather than histological examination with specific staining methods, such as Giemsa, which has a sensitivity and specificity of 95% and 98%, respectively, for detecting *H. pylori* [26]. Among the several issues involved in aspirin-related cancer recurrence, we would like to focus on preventing bleeding by avoiding the indiscriminate use of aspirin. Further research is needed to test the non-inferiority (or equivalence) of aspirin use. Nevertheless, this is the only study to confirm the chemopreventive effect of aspirin alone by evaluating *H. pylori*-negative patients. Although the difference was not statistically significant, the recurrence rate of MGC was higher in the aspirin non-user group, and this effect was not influenced by the duration of aspirin use. 

## 5. Conclusions

This study showed that aspirin was not associated with a lower risk of MGC in *H. pylori*-negative patients. Therefore, clinicians must consider the risk of bleeding complications and not administer aspirin prophylactically to reduce the risk of MGC after ESD. Future prospective studies are required to better understand the effects of aspirin on metachronous recurrence.

## Figures and Tables

**Table 1:** Baseline characteristics of the study cohort.

Variables	Total Patients (n = 2151)	Aspirin Users (n = 236)	Aspirin Nonusers (n = 1915)
Age, years	63.9 ± 9.5	68.5 ± 8.8	63.4 ± 9.5
Male	1698 (78.9)	211 (89.4)	1487 (77.7)
Tumor size, mm	12.0 (8.0–19.0)	12.0 (8.0–18.0)	12.0 (8.0–19.0)
Tumor location			
Lower	1355 (63.0)	150 (63.6)	1205 (62.9)
Middle	749 (34.8)	82 (34.7)	667 (34.8)
Upper	47 (2.2)	4 (1.7)	43 (2.2)
Tumor macroscopic type			
Elevated	268 (12.5)	33 (14.0)	235 (12.3)
Flat	310 (14.4)	36 (15.3)	274 (14.3)
Depressed	1009 (46.9)	123 (52.1)	889 (46.3)
Mixed	564 (26.2)	44 (18.6)	520 (27.2)
Depth of invasion			
Mucosa (T1a)	1961 (91.2)	212 (89.8)	1749 (91.3)
Submucosa (T1b)	144 (6.7)	16 (6.8)	128 (6.7)
Histologic differentiation			
Differentiated	2146 (99.8)	236 (100)	1 910 (99.7)
Undifferentiated	5 (0.2)	0	5 (0.3)
Histologic heterogeneity			
Absent	2009 (93.4)	219 (92.8)	1790 (93.5)
Present	142 (6.6)	17 (7.2)	125 (6.5)
Lymphovascular invasion	44 (2.0)	7 (3.0)	37 (1.9)
Comorbidities			
Hypertension	378 (17.6)	169 (71.6)	209 (10.9)
Diabetes mellitus	240 (11.2)	92 (39.0)	148 (7.7)
Myocardial infarction	32 (1.5)	16 (6.8)	16 (0.8)
Heart failure	66 (3.5)	44 (32.8)	22 (1.3)
Chronic kidney disease	87 (4.0)	29 (12.3)	58 (3.0)
Liver cirrhosis	56 (2.6)	13 (5.5)	43 (2.2)
Cerebrovascular	206 (9.6)	91 (38.6)	115 (6.0)
Comorbidities (≥2)	225 (12.0)	81 (69.8)	142 (8.2)
Metformin	199 (9.3)	52 (22.0)	147 (7.7)

Abbreviation: T1a, gastric cancer with intramucosal; T1b, gastric cancer with submucosal.

**Table 2:** Factors associated with metachronous gastric cancer.

Factors	Univariable Analysis		Multivariable Analysis	
	HR (95% CI)	*p*-Value	HR (95% CI)	*p*-Value
Age (per year)	1.04 (1.02–1.05)	<0.001	1.04 (1.02–1.05)	<0.001
Male sex	1.20 (0.81–1.80)	0.36		
Tumor size, mm	1.01 (0.99–1.03)	0.077	1.01 (0.99–1.02)	0.47
Tumor location				
Lower	Reference		Reference	
Middle	1.32 (0.96–1.81)	0.080	1.33 (0.96–1.82)	0.082
Upper	1.06 (0.34–3.35)	0.92	0.95 (0.30–3.00)	0.93
Tumor macroscopic type				
Elevated	Reference		Reference	
Flat	0.71 (0.40–1.25)	0.24	0.76 (0.43–1.35)	0.35
Depressed	0.64 (0.41–1.02)	0.062	0.75 (0.47–1.19)	0.23
Mixed	0.92 (0.57–1.48)	0.72	0.99 (0.62–1.61)	0.98
Depth of invasion				
Mucosa (T1a)	Reference			
Submucosa (T1b)	0.94 (0.52–1.70)	0.84		
Histologic differentiation				
Differentiated	Reference			
Undifferentiated	0.05 (0.00–44,708.22)	0.67		
Histologic heterogeneity				
Absent	Reference			
Present	1.49 (0.89–2.50)	0.13		
Lymphovascular invasion	1.40 (0.52–3.78)	0.51		
Comorbidities				
Hypertension	0.86 (0.58–1.27)	0.44		
Diabetes mellitus	1.19 (0.77–1.85)	0.43		
Myocadial infarction	0.68 (0.17–2.73)	0.59		
Heart failure	1.08 (0.51–2.31)	0.84		
Chronic kidney disease	0.97 (0.48–1.99)	0.94		
Liver cirrhosis	1.16 (0.51–2.63)	0.72		

Abbreviation: T1a, gastric cancer with intramucosal; T1b, gastric cancer with submucosal.

**Table 3:** Association between duration of use of aspirin and gastric cancer.

Factors	Univariable Analysis	
	HR (95% CI)	*p*-Value
Duration		
Non-aspirin use	Reference	
>6 months	1.15 (0.73–1.79)	0.55
>1 years	1.22 (0.78–1.93)	0.38
>2 years	1.26 (0.78–2.01)	0.34
>3 years	1.16 (0.69–1.95)	0.57
>4 years	1.15 (0.66–2.00)	0.61
>5 years	1.01 (0.54–1.86)	0.98
Duration		
Non-aspirin use	Reference	
≤1 years	0.64 (0.20–2.01)	0.45
1–4 years	1.35 (0.66–2.76)	0.41

## Data Availability

The data presented in this study are available upon request from the corresponding author. The data are not publicly available because of privacy and ethical restrictions.

## Supplementary Materials

The following supporting information can be downloaded at: https://www.mdpi.com/article/10.3390/jcm11010193/s1, Figure S1: Cumulative incidence of metachronous gastric cancer according to aspirin use on *H. pylori* positive groups, Table S1: Subgroup analysis of factor associated with metachronous gastric cancer.
