# Supplementary material for: Aspirin Use Is Not Associated with the Risk of Metachronous Gastric Cancer in Patients without Helicobacter pylori Infection
Source: J Clin Med. 2021 Dec 30;11(1):193. doi: 10.3390/jcm11010193 (PMC8745470; doi:10.3390/jcm11010193)
Supplement: Supplementary file 1 [file jcm-11-00193-s001.zip › jcm-1474754-supplementary.pdf]

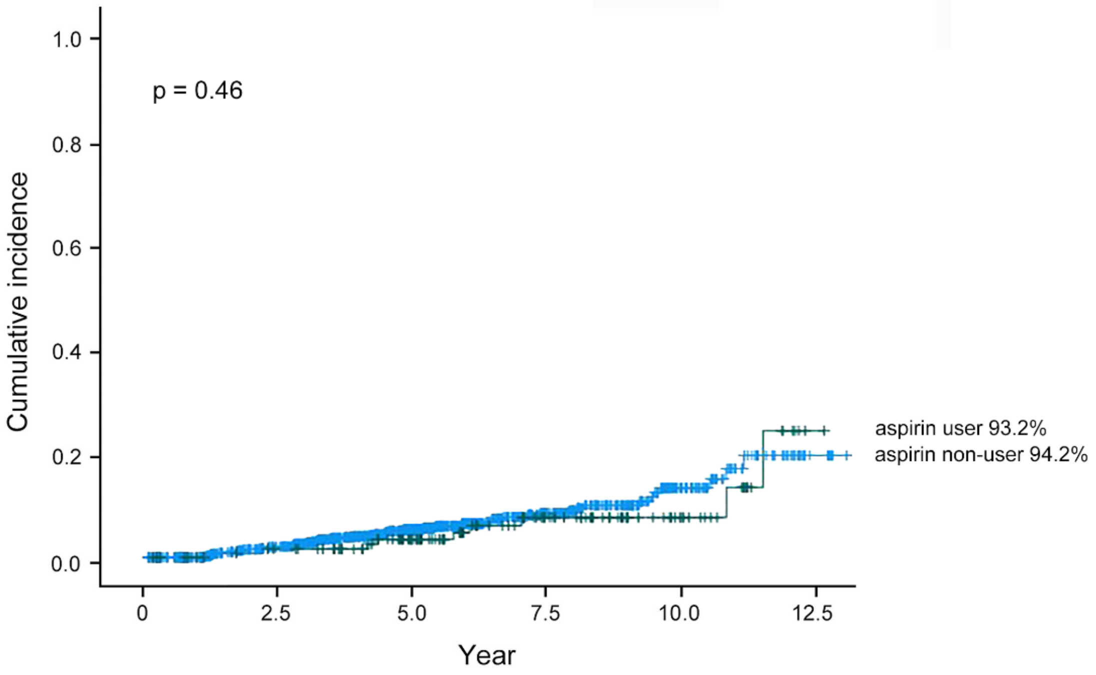

Supplementary Materials Figure S1. Cumulative incidence of metachronous gastric cancer according to aspirin use on *H.pylori* positive groups.

Supplementary Materials Table S1. Subgroup analysis of factor associated with metachronous gastric cancer

Subgroup	Aspirin users (n = 236)	Aspirin nonusers (n = 1,915)	HR (95% CI)	*P for interaction*
**Age**				0.17
< 60 years	41	646	0.24 (0.03-1.77)	
≥ 60 years	195	1,269	1.20 (0.76-1.87)	
**Sex**				0.84
Female	25	428	1.10 (0.26-4.68)	
Male	211	1,487	1.09 (0.69-1.71)	
**Tumor size**				0.33
< 20	178	1,439	1.24 (0.76-2.02)	
≥ 20	58	476	0.76 (0.30-1.95)	
**Histologic differentiation**				-
Differentiated	236	1,910	1.11 (0.72-1.70)	
Undifferentiated	0	5	-	
